# Development and validation of an interpretable machine learning model to predict malignant cerebral edema after endovascular treatment in acute anterior circulation large vessel occlusion stroke

**DOI:** 10.3389/fneur.2025.1694030

**Published:** 2026-01-02

**Authors:** Zhiwei Dong, Zhengyu Huang, Hui Hu, Yanan Wang, Chenxin Jiang, Gang Li, Feifeng Liu, Tianrui Zhu, Hao Shen, Chen Chen, Yue Zhang

**Affiliations:** 1 Department of Neurology, Shanghai East Hospital, School of Medicine, Tongji University, Shanghai, China; 2 Department of Neurology, West China Hospital, Sichuan University, Chengdu, China

**Keywords:** acute ischemic stroke, machine learning, malignant cerebral edema, predictive model, SHapley Additive exPlanations

## Abstract

**Background:**

Malignant cerebral edema (MCE) is a life-threatening complication following endovascular treatment (EVT) in patients with acute anterior circulation large vessel occlusion (LVO) stroke. This study aimed to develop and validate a machine learning (ML)-based predictive model for early risk assessment of MCE in this population.

**Methods:**

We retrospectively collected data of 364 acute ischemic stroke patients with acute anterior circulation large vessel occlusion from a comprehensive stroke center in Shanghai, between August 2018 and December 2024. Eighty percent of patients were randomly assigned to the training set, and the remaining 20 % to the internal validation set. Additional 162 patients from the One Pass Tirofiban In Management of Ischemic Stroke Thrombectomy In China (OPTIMISTIC) trial were included as an external validation set. Six machine learning models were developed, and the model with the highest area under the receiver operating characteristic curve (AUC) was selected as the optimal model. Its performance was evaluated in both internal and external validation sets. Decision curve analysis (DCA) and calibration curves were plotted to assess clinical utility. SHapley Additive exPlanations (SHAP) was employed to perform interpretative analysis of the model.

**Results:**

In this study, a total of 79 patients developed malignant cerebral edema (MCE), including 45 out of 291 (15.46%) patients in the training set, 13 out of 73 (17.81%) patients in the internal validation set, and 21 out of 162 (12.96%) patients in the external validation set. The random forest model performed best, achieving an AUC of 0.901 (95% CI: 0.858–0.943) in the training set, 0.849 (95% CI: 0.700–0.970) in the internal validation set, and 0.724 (95% CI: 0.606–0.841) in the external validation set.

**Conclusion:**

This study developed and externally validated an interpretable machine learning model to predict the risk of MCE in patients with acute anterior circulation LVO stroke following EVT.

## 1 Introduction

Stroke is one of the leading causes of death and disability globally (1). Endovascular thrombectomy (EVT) has emerged as a safe and effective treatment for patients with large vessel occlusion (LVO) stroke in the anterior circulation (2, 3). However, despite successful recanalization, approximately 45% of patients continue to experience poor clinical outcomes after the procedure (4, 5). This phenomenon is referred to as futile recanalization. The underlying mechanisms of futile recanalization have not been fully elucidated, but it is thought to be partially associated with the development of cerebral edema caused by ischemia–reperfusion injury. Malignant cerebral edema (MCE) is a severe complication of LVO stroke and constitutes a major cause of unfavorable prognosis, with reported mortality rates as high as 80% (6–8). Although therapeutic options for MCE are limited, previous randomized controlled trials have demonstrated that early decompressive hemicraniectomy can significantly reduce mortality and improve the likelihood of favorable functional outcomes (9–11). Therefore, timely identification and accurate prediction of MCE are critical for guiding clinical decision-making and improving patient prognosis.

Recent studies have developed models to predict MCE risk, initially relying on traditional statistical methods like logistic regression and nomograms (12, 13). More recent advances include machine learning algorithms like random forests, support vector machines, and gradient boosting trees (14, 15), which better capture complex nonlinear relationships (16) but often lack transparency, hindering clinical adoption. Application of machine learning for predicting MCE in acute anterior circulation LVO stroke patients undergoing EVT remains limited, mainly focusing on radiomic features from imaging (17), rather than clinical parameters. These studies are often constrained by small sample sizes and lack of external validation, raising concerns about their generalizability and practical utility. Against this backdrop, we sought to develop a simple, reliable, and interpretable clinical model to enable early identification of patients at high risk for MCE, aiming to bridge this important gap and support timely intervention.

In this study, we selected six well-established machine learning algorithms: Random Forest, Decision Tree, Multilayer Perceptron, Logistic Regression, Naive Baye, and K-Nearest-Neighbors, with the aim of developing and validating an interpretable machine learning model.

## 2 Materials and methods

### 2.1 Ethics statement

This retrospective cohort study was approved by the Ethics Committee of Shanghai East Hospital, Tongji University (approve number: 2024–016). It was conducted by the principles of the Declaration of Helsinki.

### 2.2 Study population

Derivation sample. We consecutively reviewed patients with acute anterior circulation LVO stroke treated with EVT at Shanghai East Hospital, Tongji University between August 2018 and December 2024. This comprehensive stroke center in Eastern China maintains a standard EVT workflow with more than 100 EVT cases per year. The inclusion criteria were as follows: (1) age ≥ 18 years; (2) patients with acute ischemic stroke within 24 h of onset; (3) non-contrast head computed tomography (CT) performed before EVT to exclude intracranial hemorrhage; (4) pre-treatment CT perfusion (CTP) imaging available; (5) digital subtraction angiography (DSA) confirming occlusion of the internal carotid artery (ICA) or middle cerebral artery (MCA), with successful recanalization defined as a modified Thrombolysis in Cerebral Infarction (mTICI) score of 2b–3 following EVT; and (6) dynamic head CT performed within 5 days after EVT to assess edema. Exclusion criteria were incomplete clinical or imaging data.

Validation sample. Data from the One Pass Tirofiban In Management of Ischemic Stroke Thrombectomy In China (OPTIMISTIC) trial, led by Shanghai East Hospital, were included as an external validation set (18). The OPTIMISTIC trial is a multicenter, prospective, open-label, blinded endpoint assessment phase II randomized controlled clinical trial, recruiting patients from seven stroke centers in China, aiming to evaluate whether intravenous tirofiban administration prior to EVT could improve the efficacy of the procedure by achieving first-pass reperfusion without increasing the risk of intracranial hemorrhage. All data for this study were obtained from patients with acute anterior circulation large vessel occlusion stroke, excluding those recruited from Shanghai East Hospital to avoid duplication. All imaging scans in validation set were re-evaluated in this study to enhance the stability of imaging parameters with permission of corresponding author of the OPITMISTIC.

### 2.3 Data collection

The following data were collected: (1) Demographics: age, sex, history of smoking; (2) Medical history: hypertension, diabetes mellitus, ischemic stroke, coronary artery disease; (3) Perfusion imaging parameters: infarct core volume and ischemic penumbra volume; (4) Interventional and procedure-related characteristics: pre-treatment NIHSS score, onset-to-puncture time, occlusion site, number of retrieval attempts, type of thrombectomy procedure; (5) Laboratory findings: red blood cell count (RBC), hemoglobin (Hb), white blood cell count (WBC), platelet count (PLT), neutrophil count, creatinine (CREA), urea, uric acid (UA), prothrombin time (PT), international normalized ratio (INR), fibrinogen (FIB), thrombin time (TT), glucose, potassium, sodium.

### 2.4 Neuroimaging

All patients underwent a baseline non-contrast CT scan after admission and before EVT. Alberta Stroke Program Early CT Score (ASPECTS) was evaluated initially. Within 24 h following EVT, a follow-up non-contrast CT scan was performed for all patients. Additionally, some patients received a second follow-up non-contrast CT scan within 5 days after thrombectomy if clinically needed. Baseline CT imaging also included brain CTP and computed tomography angiography (CTA). The axial coverage ranged from 80 to 160 mm. All the CTP parameters both in derivation and validation set were processed by commercial software MIStar (Apollo Medical Imaging Technology, Melbourne, Vic, Australia), including cerebral blood flow and delay time Total ischemic lesion and ischemic core were defined by delay time≥3 s, and cerebral blood flow ≤30%, respectively. The mismatch ratio was defined as the volume of the total ischemic lesion divided by the volume of the ischemic core. Hypoperfusion intensity ratio(HIR) was used for the evaluation of collateral status (19).

### 2.5 Definition of MCE

Cerebral edema was classified according to the Safe Implementation of Thrombolysis in Stroke-Monitoring Study (SITS-MOST) protocol (20), as no swelling (0); brain swelling comprising <1/3 of the hemisphere (1); swelling comprising >1/3 of the hemisphere (2) or midline shift (3); We then defined grade 3 as MCE (21). With the permission from the OPITMISTIC corresponding author, all CT scans from the derivation set and validation set were evaluated by the same criteria. In derivation set, MCE was evaluated by CT scans within 5 days after onset, and the one most closed to 5 days was used if multiple scans conducted. In validation set, brain imaging was collected 24–72 h (± 8 h) after onset.

### 2.6 Statistical analysis and model development

Patients from the derivation sample were randomly divided into a training set and an internal validation set in an 8:2 ratio. Continuous variables were shown as means with standard deviations (SD) or medians with interquartile ranges (IQR) based on the distribution. Median imputation was applied to complete missing data if less than 5% by random. Categorical variables were shown as counts with percentages. In univariate analysis, continuous variables were analyzed using a Student’s t-test if normally distributed and a Wilcoxon ranked sum test if non-normal. Categorical variables were analyzed using Pearson’s *χ*^2^-test or Fisher’s exact test if a small number. To ensure model stability and interpretability, we assessed multicollinearity among all candidate predictors prior to feature selection. Variables with a variance inflation factor (VIF) greater than 5 were considered to have high multicollinearity and were excluded from the subsequent feature selection process. Standardized mean differences (SMD) were additionally calculated to assess baseline balance, with SMD < 0.1 indicating negligible imbalance.

We used the least absolute shrinkage and selection operator (LASSO) regression and recursive feature elimination (RFE) to select relevant candidate features. Six machine learning algorithms - Random Forest, Decision Tree, Multilayer Perceptron, Logistic Regression, Naive Bayes, and K-Nearest-Neighbors—were trained, with hyperparameters optimized via grid search. The models with the highest area under the receiver operating characteristic (ROC) curve (AUC) during tuning were selected with an AUC value ≥ 0.7 indicating good discrimination (22). Models were trained on the training set and validated internally and externally. Performance was evaluated using AUC, calibration curves, and the Hosmer–Lemeshow test to assess agreement between predicted and actual outcomes. Decision curve analysis (DCA) was performed to evaluate clinical utility across various thresholds. To enhance interpretability, SHapley Additive exPlanations (SHAP) was used to quantify feature contributions and visualize their impact on individual predictions, providing both global and local insights into the model’s decision-making.

All statistical analyses were performed using Python software (version 3.10.6), and two-sided *p*-values less than 0.05 were considered statistically significant.

## 3 Results

### 3.1 Baseline characteristics

**Figure 1:**
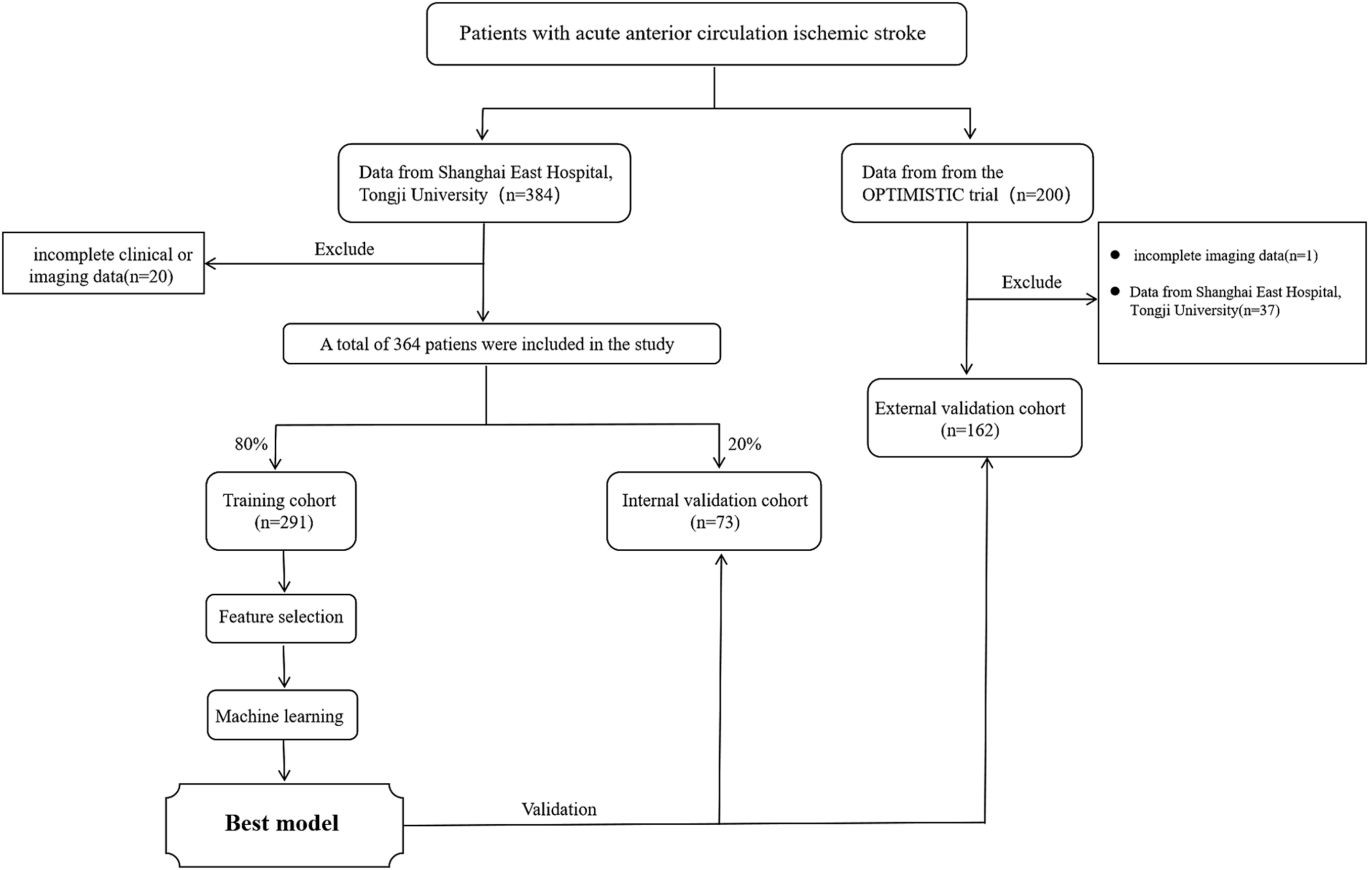
Study flowchart of the study design.

Figure 1 showed 364 patients from Shanghai East Hospital were included as a derivation cohort, of which 291 were randomly allocated into the training cohort and 73 were in the internal validation cohort. After excluding 37 patients from Shanghai East Hospital, Tongji University, 162 patients from the OPTIMISTIC trial were included in the external validation cohort.

Table 1 showed the baseline characteristics of patients with acute anterior circulation LVO stroke who underwent EVT. The baseline characteristics between the training and internal validation sets were generally balanced and comparable. In this study, a total of 75 patients developed MCE, including 45 out of 291 (15.46%) patients in the training set, 13 out of 73 (17.81%) patients in the internal validation set, and 21 out of 162 (12.96%) patients in the external validation set.

**Table 1:** Baseline characteristics of the participants.

Characteristic	Training set (*N* = 291)	Internal validation set (*N* = 73)	*p*-value	External validation set (*N* = 162)
Outcome				
MCE	45/291 (15.46%)	13/73 (17.81%)	0.625	21/162 (12.96%)
Demographics				
Age (years)	71.00 (65.00–77.00)	70.00 (63.00–78.00)	0.507	66.00 (57.00–72.00)
Female, *n* (%)	112/291 (38.49%)	31/73 (42.47%)	0.534	43/162 (26.54%)
Smoker, *n* (%)	103/291 (35.40%)	16/73 (21.92%)	0.028	35/162 (21.60%)
Medical history, *n* (%)				
Hypertension	196/291 (67.35%)	44/73 (60.27%)	0.254	84/162 (51.85%)
Diabetes mellitus	83/291 (28.52%)	23/73 (31.51%)	0.616	25/162 (15.43%)
Ischemic stroke	65/291 (22.34%)	16/73 (21.92%)	0.939	22/162 (13.58%)
Coronary artery disease	40/291 (13.75%)	9/73 (12.33%)	0.751	9/162 (5.56%)
Perfusion imaging, mL				
Infarct core volume	16.00 (5.00–42.00)	20.00 (6.00–50.00)	0.301	9.00 (3.00–23.75)
Ischemic penumbra volume	96.00 (62.70–133.00)	100.00 (64.00–137.00)	0.913	95.00 (56.25–131.75)
Interventional surgical-related characteristics				
Pre-treatment NIHSS score	14 (10–18)	14 (1–17)	0.798	11 (8.00–15)
Onset-to-puncture time (hours)	7.00 (5.00–10.18)	6.00 (4.23–12)	0.263	12.44 (7.40–16.49)
Occlusion site, *n* (%)			0.764	
M1	160/291 (54.98%)	43/73 (58.90%)		103/162 (63.58%)
M2	31/291 (10.65%)	6/73 (8.22%)		10/162 (6.17%)
ICA	100/291 (34.37%)	24/73 (32.88%)		49/162 (30.25%)
Number of retrieval attempts, *n* (%)			0.941	
1–2	194/291 (66.67%)	49/73 (67.12%)		140/162 (86.42%)
>2	97/291 (33.33%)	24/73 (32.88%)		22/162 (13.58%)
Type of thrombectomy procedure, *n* (%)			0.975	
Thrombectomy alone	172/291 (59.11%)	43/73 (58.90%)		142/162 (87.65%)
Rescue therapy	119/291 (40.89%)	30/73 (41.10%)		20/162 (12.35%)
Laboratory findings				
RBC × 10^12^/L	4.07 (0.65)	3.97 (0.61)	0.169	4.69 (0.62)
Hb(g/L)	139 0.00(126.00–149.00)	133.00 (121.00–147.00)	0.173	141 0.00(129.00–152.00)
WBC × 10^9^/L	9.24 (7.38–11.44)	8.95 (7.30–11.01)	0.788	8.03 (6.61–10.21)
Neutrophil× 109/L	7.39 (5.77–9.61)	7.48 (5.71–8.97)	0.679	5.23 (4.24–7.85)
PLT × 10^9^/L	191.00 (155.00–240.50)	179.00 (151.00–218.00)	0.146	224.00 (177.00–265.00)
CREA (μmol/L)	70.00 (56.00–90.50)	69.00 (57.00–86.00)	0.917	71.25 (60.80–85.00)
Urea (mmol/L)	4.82 (3.89–6.29)	5.20 (4.40–6.02)	0.193	5.40 (4.40–6.69)
UA (μmol/L)	299.22 (102.40)	306.15 (106.18)	0.661	378.69 (95.71)
PT (s)	10.2 (9.02–12.10)	11.00 (9.30–12.20)	0.340	12.55 (11.63–13.30)
INR	1.03 (0.99–1.10)	1.04 (0.99–1.09)	0.721	1.02 (0.98–1.08)
FIB(g/L)	2.80 (2.36–3.47)	2.66 (2.18–3.22)	0.186	3.29 (2.78–3.67)
TT(s)	17.80 (16.30–19.20)	17.90 (16.50–19.70)	0.569	17.00 (15.43–17.90)
Glucose (mmol/L)*	7.49 (6.43–9.30)	7.01 (5.80–10.70)	0.527	7.10 (5.90–8.40)
Potassium (mmol/L)*	3.79 (0.48)	3.85 (0.51)	0.452	3.96 (0.45)
Sodium (mmol/L)*	139.00 (137.00–141.00)	139.00 (137.00–141.00)	0.508	139.90 (137.73–141.50)

Data are n, n/N (%), mean (SD), or median (IQR). SD denotes standard deviation. IQR denotes interquartile range. MCE, malignant cerebral edema; NIHSS, National Institutes of Health Stroke Scale; RBC, red blood cell count; Hb, hemoglobin; WBC, white blood cell count; PLT, platelet count; CREA, creatinine; UA, uric acid; PT, prothrombin time; INR, international normalized ratio; FIB, fibrinogen; TT, thrombin time.

*Missing data: in derivation set (Training set + Internal Validation set), glucose (2 missing), potassium (8 missing), sodium (8 missing).

Baseline characteristics of the derivation and external validation cohorts are summarized in Supplementary Table S1. Patients from external validation set were younger, with less comorbidities, and less stroke severity. Significant differences were observed across the majority of variables (several SMDs >0.2), reflecting the distinct populations due to different inclusion criteria. In multicollinearity assessment, HIR (VIF 5.089) and ASPECTS (VIF 10.8) were excluded before feature selection. In the derivation set, 70.05% of patients had two follow-up CT scans, compared to 20.99% in the external set. Nevertheless, the median time from onset to follow-up CT for MCE evaluation was similar (28.74 h vs. 36.17 h; SMD = 0.141), indicating minimal variation.

### 3.2 Feature selection

**Figure 2:**
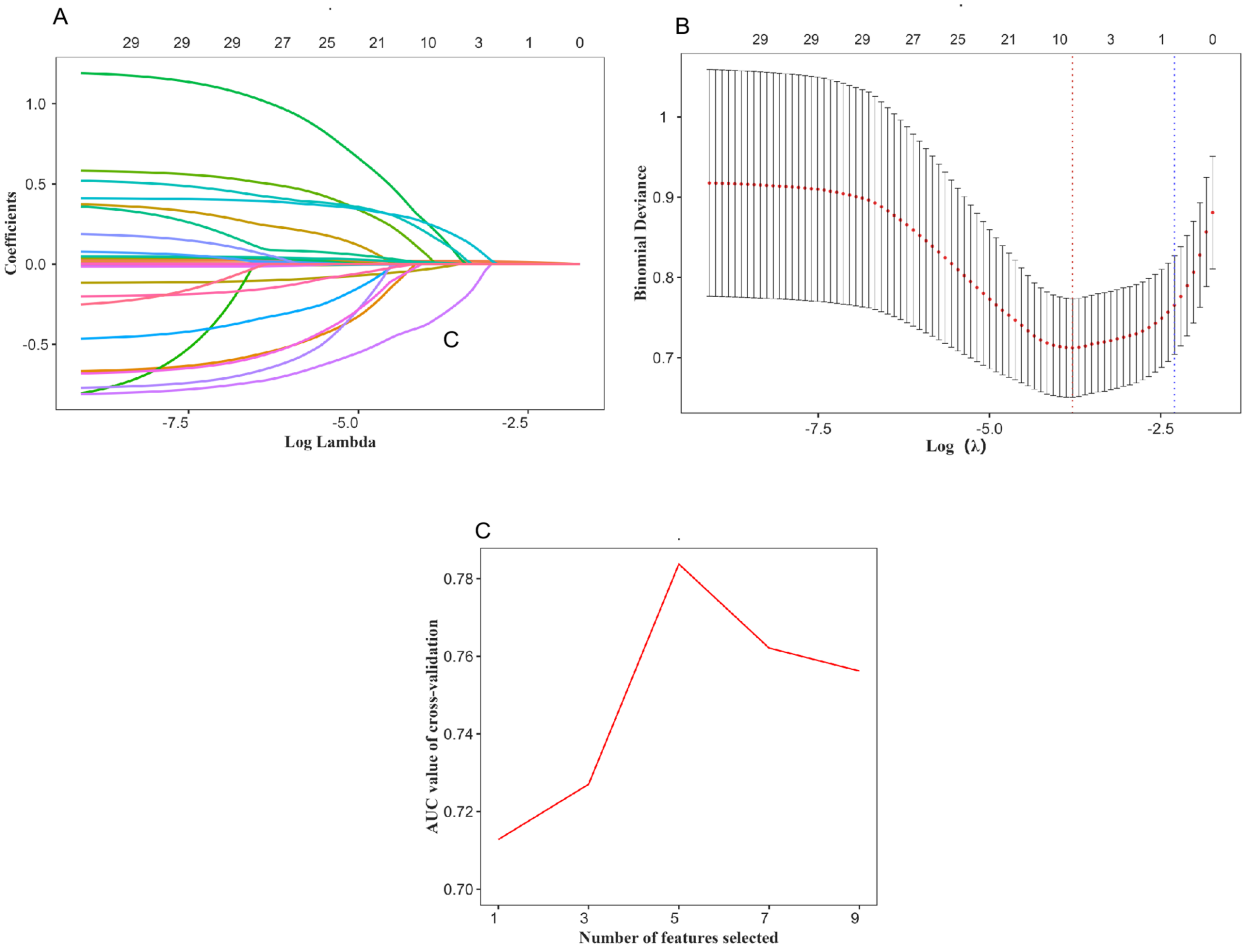
Using the LASSO regression and REF method to identify the optimal variables. **(A)** Variation characteristics of variable coefficients; **(B)** The process of selecting the optimal value of the parameter *λ* in the LASSO regression model is carried out by the cross-validation method. **(C)** Five variables were selected for MT.

Relevant features were selected from the training set using a two-step approach. First, LASSO regression was applied. As the regularization parameter log(*λ*) increased, the regression coefficients of the variables gradually approached zero (Figure 2A). A ten-fold cross-validation plot for LASSO regression was generated (Figure 2B), and the optimal value of λ (λmin) was selected, resulting in nine candidate predictive variables. Second, to further simplify the feature set and improve model stability, RFE was performed on the variables selected by LASSO. As shown in Figure 2C, five features were ultimately retained as optimal predictors and included in the machine learning models. These five features were age, infarct core volume, pre-treatment NIHSS score, occlusion site, and number of retrieval attempts.

### 3.3 Development and comparison of prediction models

**Figure 3:**
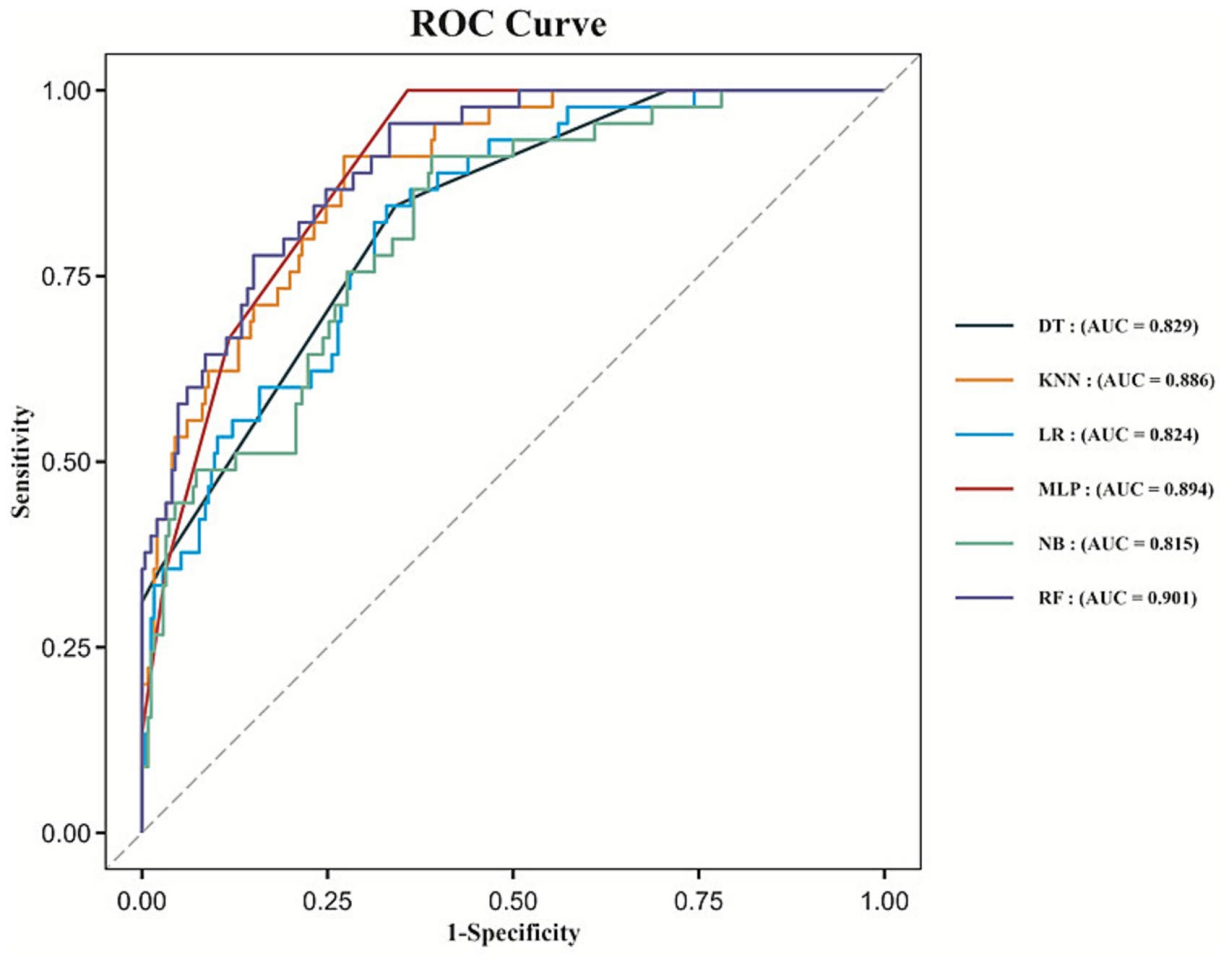
ROC curves for the machine learning models.

We developed six machine learning models to predict the risk of malignant cerebral edema following EVT in patients with acute anterior circulation LVO stroke. ROC curves for all six models are shown in Figure 3. All models demonstrated good discriminative ability, with the Random Forest model achieving the highest AUC of 0.901 (95% CI: 0.858–0.943). The AUCs for the Decision Tree, Multilayer Perceptron, Logistic Regression, Naive Bayes, and K-Nearest-Neighbors models were 0.829 (95% CI: 0.772–0.885), 0.894 (95% CI: 0.857–0.931), 0.824 (95% CI: 0.763–0.885), 0.815 (95% CI: 0.751–0.879) and 0.886 (95% CI: 0.839–0.932), respectively. Based on the highest AUC value, the Random Forest model was selected as the optimal model.

**Figure 4:**
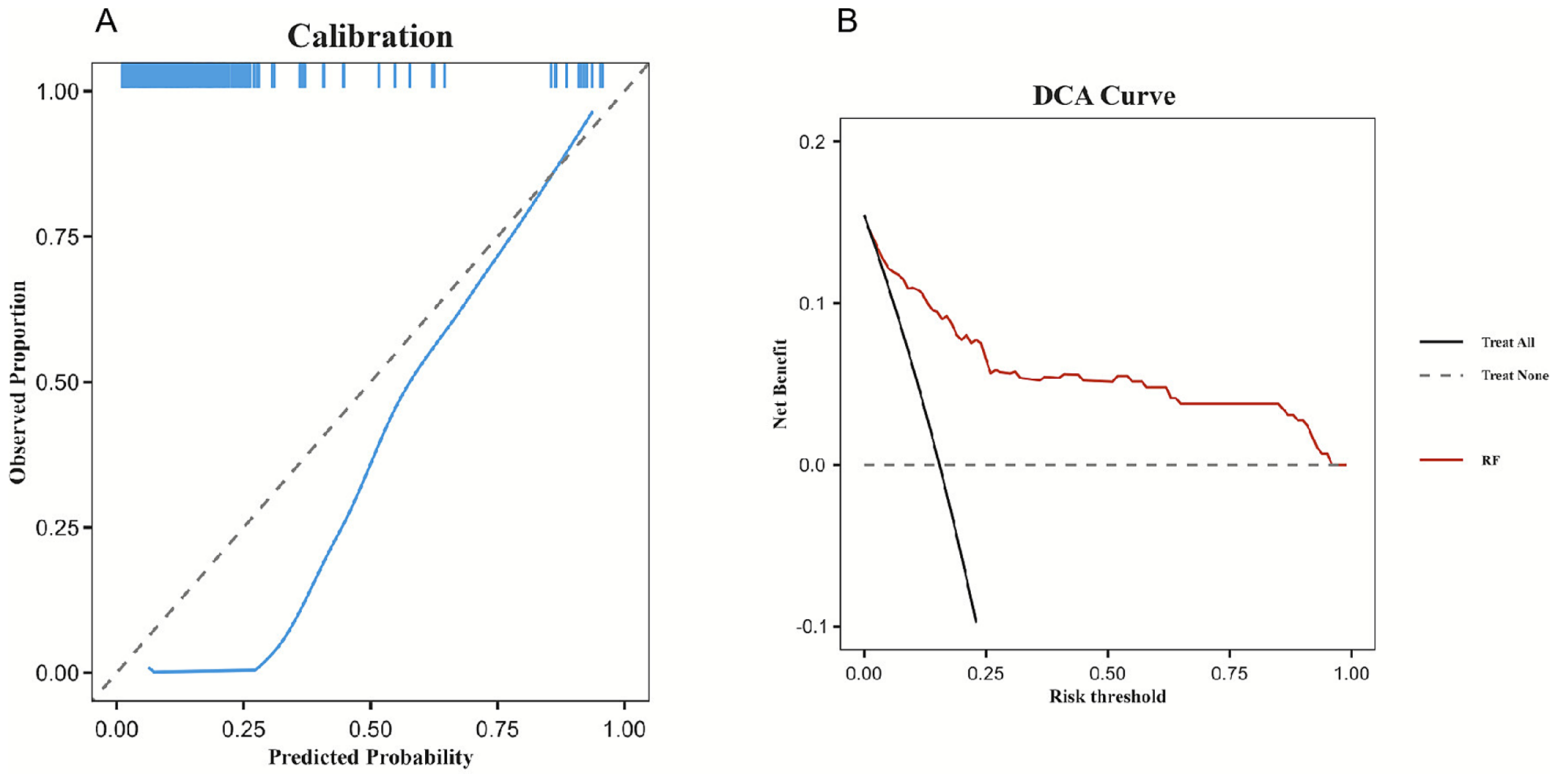
Calibration capability and clinical benefit of the model. **(A)** Calibration curve of the Random Forest model. **(B)** DCA of Random Forest the model.

Calibration curves for all six models are presented in Figure 4A, with the Random Forest model showing good calibration (Hosmer–Lemeshow test, *p* = 0.338). In terms of clinical utility, the Random Forest model demonstrated robust net benefit across a wide range of threshold probabilities, as illustrated in the DCA (Figure 4B).

### 3.4 Model validation

**Figure 5:**
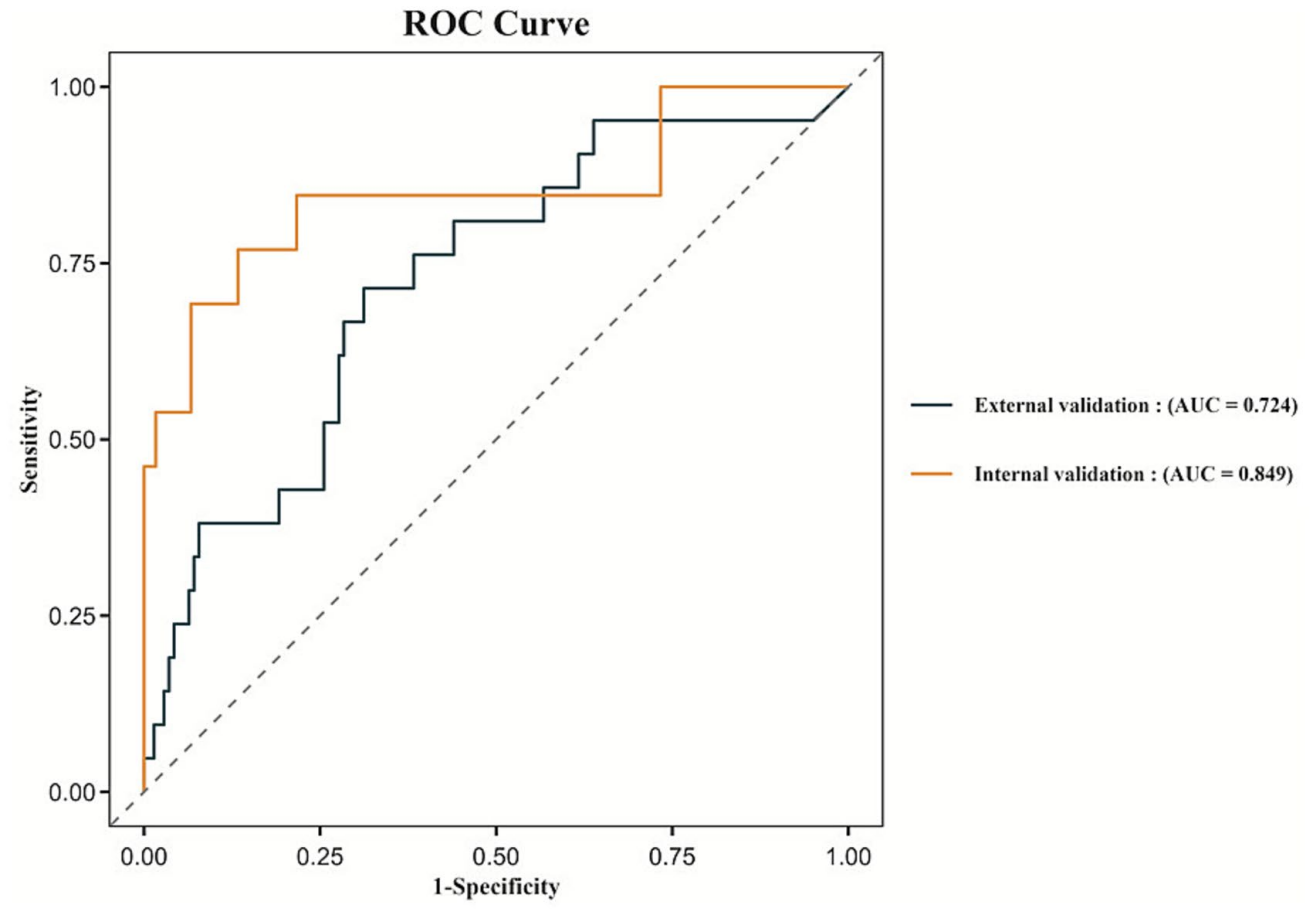
ROC curves for the internal validation and external validation.

The performance of the Random Forest model remained stable across both internal and external validation sets. In the internal validation set, the ROC curve yielded an AUC of 0.849 (95% CI: 0.700–0.970), while in the external validation set, the AUC was 0.724 (95% CI: 0.606–0.841), as shown in Figure 5. These findings indicate that the model possesses good generalizability and external applicability.

### 3.5 Model interpretation

To enhance model interpretability, we applied SHAP to quantify the contribution of each predictor to the model output. This method enables both global and individual-level interpretation of the model, thereby facilitating its application in clinical settings.

**Figure 6:**
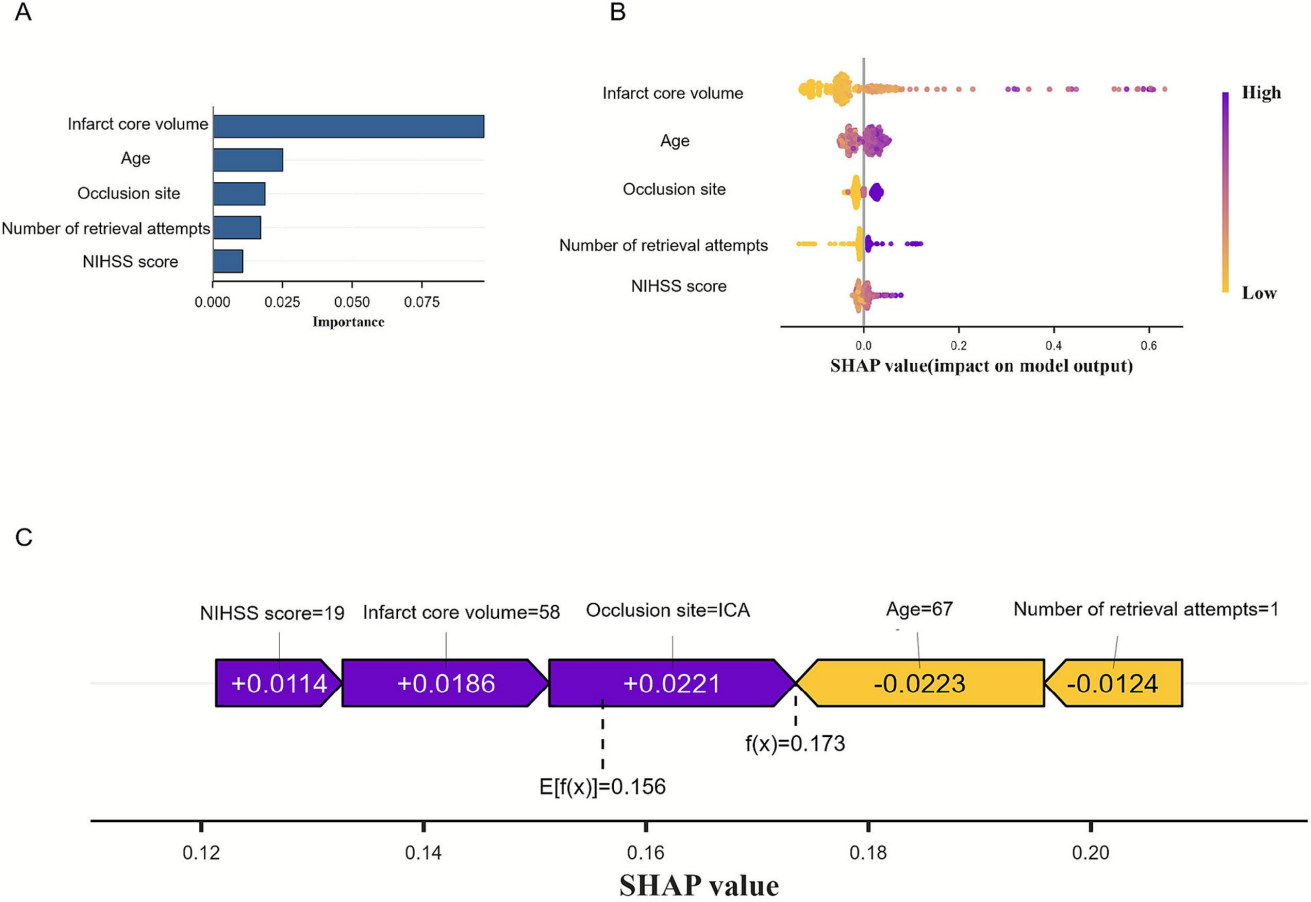
Visually interpret machine learning models using SHAP. **(A)** SHAP summary bar plot. **(B)** The SHAP summary dot plot. **(C)** SHAP force plot.

At the global level, the SHAP summary bar plot (Figure 6A) displays the average absolute SHAP values of the selected features in descending order, reflecting their overall contribution to the model. Among the five predictors, infarct core volume had the greatest influence on the model output, followed by age, number of retrieval attempts, occlusion site, and pre-treatment NIHSS score.

The SHAP summary dot plot (Figure 6B) illustrates how feature values influence predictions. Each dot represents a patient’s value for a feature-purple for high, and yellow for low-showing that larger infarct core volume correlated with higher SHAP values, indicating increased MCE risk. This visualization reveals how individual feature contributions impact model outputs. The SHAP force plot (Figure 6C) details each feature’s contribution for a specific patient: longer bars and purple color indicate a positive impact, while shorter and yellow bars suggest a negative effect. For example, an NIHSS score of 19, infarct volume of 58 mL, and ICA occlusion raised the risk, whereas an age of 67 and one retrieval attempt lowered it. The predicted risk (f(x) = 0.173) was slightly above the model’s average output (E[f(x)] = 0.156), suggesting a moderately elevated risk of MCE for this patient.

## 4 Discussion

This study aimed to develop a predictive model for MCE in patients with acute anterior circulation LVO stroke after EVT. Six machine learning algorithms were employed to construct the model. Among them, the Random Forest algorithm demonstrated the best performance, exhibiting good discriminative ability and calibration, as well as higher net clinical benefit in DCA. The model’s robustness and generalizability were further confirmed by external validation. Finally, we applied the SHAP method to quantify the contributions of each feature and visually illustrate the model’s decision-making process. Moreover, the reduced performance in the external validation set also warrants clarification. The external validation set differed substantially from the derivation set in key predictors such as age, pre-treatment NIHSS score, infarct core volume, and number of retrieval attempts, and these variables carry the greatest weight in our model; thus, case-mix differences likely contributed to the lower external AUC. In addition, unlike the single-center derivation set, the external set involved six centers with heterogeneous operator experience and peri-procedural management, which may have influenced the occurrence of MCE and modestly reduced model discrimination without indicating model failure. The different time window of MCE evaluation might also affect the model performance.

Several previous studies have developed predictive models for MCE using traditional logistic regression (12, 13), including a nomogram with eight clinical features, including the Glasgow Coma Scale (GCS) score, baseline NIHSS score, and ASPECTS (23), which were well-validated predictors. However, variables such as infarct core volume and occlusion site, have rarely been included systematically. In our model, infarct core volume was the most important predictor, significantly improving performance and highlighting its clinical value for early risk stratification for MCE. Existing nomograms for MCE prediction rely on linear assumptions and population-level coefficients, which limit their ability to account for nonlinear effects or to generate patient-specific explanations. More importantly, nomograms cannot quantify how individual predictors contribute to the risk estimate for a specific patient. While machine learning algorithms have gained popularity for their ability to capture complex, non-linear relationships in high-risk populations (16). Recent studies have explored various machine learning and deep learning approaches for predicting MCE after ischemic stroke and have reported high discriminative performance. For example, a two-center study combining clinical variables with CT-based radiomic features achieved a validation AUC of approximately 0.92 (24), and another MRI radiomics model similarly yielded an external validation AUC of about 0.84 (17). However, these models mostly rely on advanced imaging processing techniques, or their generalizability is limited by relatively small sample sizes and the absence of external validation. In contrast, our proposed model uses only readily accessible clinical variables. Many models lack independent external validation. External validation is essential for assessing generalizability across different settings. To address this, our study incorporated an external validation set from six independent centers, strengthening the model’s clinical applicability.

Previous studies shows that larger infarct core volumes were associated with an increased risk of midline shift due to space-occupying cerebral edema (25), supporting our findings. Additionally, a higher number of retrieval attempts is significantly associated with increased risk of MCE, consistent with observations from previous studies (26, 27). Occlusion site is a known predictor of MCE (28, 29), with ICA occlusion posing a higher risk of extensive infarction and secondary cerebral edema compared to MCA occlusion, which aligns with our results. The relationship between age and MCE remains unclear. Cerebral atrophy in elderly patients may provide extra intracranial space, potentially reducing MCE risk (12), but very old patients remain at high risk of poor early outcomes after ischemic stroke (30). Conversely, higher NIHSS scores, indicating more severe neurological deficits, suggest larger infarct area and greater vascular occlusion, thereby increasing the risk of MCE. This association is supported by previous studies.

MCE is a leading cause of early mortality and disability following acute ischemic stroke. In clinical practice, the early identification of patients at high risk for MCE and the implementation of individualized management and intervention strategies are crucial for improving outcomes (31). Although numerous studies have focused on the early prediction of MCE, striking a balance between predictive performance and clinical interpretability remains a major challenge. Therefore, the development of a predictive model that combines high accuracy, interpretability, and clinical practicality represents a key direction for future research. Future research on MCE after EVT should focus on multicenter studies across diverse populations to validate model generalizability, investigate new clinical and imaging predictors to enhance performance and mechanistic insight, combine clinical data with radiomics or deep learning for more accurate and interpretable models, and evaluate the model’s practicality and impact on decision-making and outcomes in real-world clinical settings to facilitate translation into routine practice.

Our study has several strengths. First, independent external validation confirmed the model’s robustness and potential for clinical practice. Second, we used a two-stage feature selection combining LASSO regression with recursive elimination, balancing traditional variable screening with machine learning’s ability to capture complex nonlinear interactions. This reduced overfitting, improved efficiency, and, with infarct core volume as a continuous, objective variables, enhanced the precision and stability of the model. This approach enabled us to develop a reliable predictive tool despite a modest sample size, lowering costs and expanding applicability. Finally, applying SHAP provided clear insights into each feature’s global and individual contributions, improving transparency and clinical acceptance of the model (32).

This study has several limitations. First, the data were retrospectively collected from a limited number of centers, which may introduce selection bias, with limited availability to other important variables (such as peri-procedural blood pressure parameters), and affect the generalizability of the model. There were certain differences in CTP acquisition protocols across centers, and although all datasets underwent centralized quality control and standardized processing, residual measurement bias arising from upstream imaging variability cannot be fully excluded. Second, due to the absence of certain key clinical variables, such as onset-to-perfusion time peri-procedural blood pressure changes, specific thrombectomy technique, which are known to significantly influence stroke outcomes. Not all potentially relevant predictors could be included, which may limit the model’s ability to capture the full spectrum of MCE risk under varying clinical scenarios. This missing information could compromise the precision of individual-level risk assessment, particularly where complex interactions among predictors exist. We recommend that future prospective studies systematically incorporate these variables to further enhance the model’s reliability in clinical risk evaluation. In addition, although infarct core volume demonstrated high predictive value in our model, its measurement relies on advanced imaging techniques that are not widely available in all clinical settings, potentially limiting the model’s applicability in routine practice. Although tirofiban treatment was not evaluated in our model due to non-uniform administration in our set, its potential relevance warrants exploration in future stratified studies. Finally, although the model was internally and externally validated with stable performance, further prospective studies with larger, multicenter cohorts are needed to evaluate its clinical utility and generalizability.

## 5 Conclusion

This study developed and externally validated an interpretable machine learning model to predict the risk of MCE in patients with acute anterior circulation LVO stroke following EVT. The incorporation of SHAP enhanced the model’s transparency and its ability to provide individualized risk interpretation. The findings suggest that this model may assist in the early identification and management of high-risk patients. To further improve its clinical applicability, future validation and modification using large, multicenter prospective registries are warranted.

## Data Availability

The raw data supporting the conclusions of this article will be made available by the authors, without undue reservation.
