# Supplementary material for: Development and validation of an interpretable machine learning model to predict malignant cerebral edema after endovascular treatment in acute anterior circulation large vessel occlusion stroke
Source: Front Neurol. 2026 Jan 2;16:1694030. doi: 10.3389/fneur.2025.1694030 (PMC12807922; doi:10.3389/fneur.2025.1694030)
Supplement: Supplementary file 1 [file Table_1.DOCX]

**Supplementary Table 1.** Comparison of baseline characteristics between the derivation and external Validation sets

| Characteristic | Derivation set  (N=364) | External validation set  (N=162) | SMD |
| --- | --- | --- | --- |
| **Outcome** |  |  |  |
| MCE | 58 (15.93%) | 21 (12.96%) | 0.083 |
| **Demographics** |  |  |  |
| Age(years) | 70.00 (64.00-77.25) | 66.00 (57.00-72.00) | 0.399 |
| Female, n (%) | 143 (39.29%) | 43 (26.54%) | 0.267 |
| smoker, n (%) | 119 (32.69%) | 35 (21.60%) | 0.244 |
| **Medical history, n (%)** |  |  |  |
| Hypertension | 240 (65.93%) | 84 (51.85%) | 0.290 |
| Diabetes mellitus | 106 (29.12%) | 25 (15.43%) | 0.317 |
| Ischemic stroke | 81 (22.25%) | 22 (13.58%) | 0.219 |
| Coronary artery disease | 49 (13.46%) | 9 (5.56%) | 0.252 |
| **Perfusion imaging, mL** |  |  |  |
| Infarct core volume | 17.00 (5.00-44.00) | 9.00 (3.00-23.75) | 0.466 |
| Ischemic penumbra volume | 97.00 (62.85-133.25) | 95.00 (56.25-131.75) | 0.001 |
| HIR | 0.22(0.17-0.30) | - | - |
| ASPECTS | 8(6-10) | - | - |
| **Interventional surgical-related characteristics** |  |  |  |
| Pre-treatment NIHSS score | 13.76 (5.89) | 11.96 (5.05) | 0.318 |
| Onset-to-puncture time (hours) | 7.00 (5.00-11.00) | 12.44 (7.40-16.49) | 0.764 |
| Occlusion site, n (%) |  |  | 0.226 |
| M1 | 203 (55.77%) | 103 (63.58%) |  |
| M2 | 37 (10.16%) | 10 (6.17%) |  |
| ICA | 124 (34.07%) | 49 (30.25%) |  |
| Number of retrieval attempts, n (%) |  |  | 0.442 |
| 1-2 | 243 (66.76%) | 140 (86.42%) |  |
| ＞2 | 121 (33.24%) | 22 (13.58%) |  |
| Type of thrombectomy procedure, n (%) |  |  | 0.612 |
| Thrombectomy alone | 215 (59.07%) | 142 (87.65%) |  |
| Rescue therapy | 149 (40.93%) | 20 (12.35%) |  |
| **Laboratory fndings** |  |  |  |
| RBC× 10^12^/L | 4.05 (0.64) | 4.69 (0.62) | 0.999 |
| Hb(g/L) | 138 .00(125.00-149.00) | 141 .00(129.00-152.00) | 0.220 |
| WBC× 10^9^/L | 9.22 (7.34-11.26) | 8.03 (6.61-10.21) | 0.307 |
| Neutrophil× 109/L | 7.40 (5.74-9.46) | 5.23 (4.24-7.85) | 0.522 |
| PLT× 10^9^/L | 189.00 (155.00-235.00) | 224.00 (177.00-265.00) | 0.384 |
| CREA(μmol/L) | 70.00 (56.00-89.25) | 71.25 (60.80-85.00) | <0.001 |
| Urea(mmol/L) | 4.90 (3.94-6.28) | 5.40 (4.40-6.69) | 0.171 |
| UA(μmol/L) | 300.61 (103.06) | 378.69 (95.71) | 0.774 |
| PT(s) | 10.45 (9.09-12.10) | 12.55 (11.63-13.30) | 0.602 |
| INR | 1.03 (0.99-1.09) | 1.02 (0.98-1.08) | 0.183 |
| FIB(g/L) | 2.78 (2.30-3.44) | 3.29 (2.78-3.67) | 0.193 |
| TT(s) | 17.85 (16.30-19.20) | 17.00 (15.43-17.90) | 0.273 |
| Glucose (mmol/L) | 7.40 (6.23-9.53) | 7.10 (5.90-8.40) | 0.169 |
| Potassium (mmol/L) | 3.80 (0.49) | 3.96 (0.45) | 0.336 |
| Sodium (mmol/L) | 139.00 (137.00-141.00) | 139.90 (137.73-141.50) | 0.223 |
| **Follow-up brain imaging** |  |  |  |
| One follow-up scan only | 109/364(29.95%) | 128/162(79.01%) | 0.984 |
| Two follow-up scans | 255/364(70.05%) | 34/162(20.99%) | 0.984 |
| Median time from onset to evaluated scans(h) | 28.74 (20.29, 42.46) | 36.17 (28.11, 42.46) | 0.141 |

Footnote: Data are n, N (%), mean (SD), or median (IQR). SD denotes standard deviation. IQR denotes interquartile range. MCE=malignant cerebral edema; HIR= hypoperfusion intensity ratio; ASPECTS= Alberta Stroke Program Early CT Score; NIHSS=National Institutes of Health Stroke Scale; RBC=red blood cell count; Hb=hemoglobin; WBC=white blood cell count; PLT=platelet count; CREA=creatinine; UA=uric acid; PT=prothrombin time; INR=international normalized ratio; FIB=fibrinogen; TT=thrombin time.
